# Multimodal integration and stimulus categorization in putative mushroom body output neurons of the honeybee

**DOI:** 10.1098/rsos.171785

**Published:** 2018-02-21

**Authors:** Martin F. Strube-Bloss, Wolfgang Rössler

**Affiliations:** Behavioral Physiology and Sociobiology (Zoology II), Biozentrum, University of Würzburg, Am Hubland, 97074 Würzburg, Germany

**Keywords:** olfaction, vision, olfactory–visual integration, mushroom body output neurons, insects, electrophysiology

## Abstract

Flowers attract pollinating insects like honeybees by sophisticated compositions of olfactory and visual cues. Using honeybees as a model to study olfactory–visual integration at the neuronal level, we focused on mushroom body (MB) output neurons (MBON). From a neuronal circuit perspective, MBONs represent a prominent level of sensory-modality convergence in the insect brain. We established an experimental design allowing electrophysiological characterization of olfactory, visual, as well as olfactory–visual induced activation of individual MBONs. Despite the obvious convergence of olfactory and visual pathways in the MB, we found numerous unimodal MBONs. However, a substantial proportion of MBONs (32%) responded to both modalities and thus integrated olfactory–visual information across MB input layers. In these neurons, representation of the olfactory–visual compound was significantly increased compared with that of single components, suggesting an additive, but nonlinear integration. Population analyses of olfactory–visual MBONs revealed three categories: (i) olfactory, (ii) visual and (iii) olfactory–visual compound stimuli. Interestingly, no significant differentiation was apparent regarding different stimulus qualities within these categories. We conclude that encoding of stimulus quality within a modality is largely completed at the level of MB input, and information at the MB output is integrated across modalities to efficiently categorize sensory information for downstream behavioural decision processing.

## 1. Introduction

Understanding how animal brains produce a behavioural output based on combined multimodal sensory information of a prevailing scene is a crucial aim in the behavioural neurosciences. In past years, this was mainly investigated on the basis of, for instance, a distinct visual [1–3] or olfactory [4,5] sensory input. While these studies revealed insight into processing of unimodal information in the brain, in reality neuronal networks are not only confronted with single modalities at a time. To obtain reliable information about the outside world and to make efficient decisions, the neuronal machinery, for almost any behaviour, has to simultaneously analyse stimulus qualities within modalities and define the overall relevance across different sensory inputs. Honeybees, for example, can be trained to multiple sensory cues [6,7]. When foraging for nectar and pollen, bees often dive deep into flowers. In that moment, almost all ommatidia of the compound eye experience the floral colour, and odorant receptors of the antennae are heavily stimulated by the floral odour bouquet. Thus, the reward association of a nectar source has at least two major components—olfactory and visual cues—which probably is the main reason why both modalities interact in classical conditioning experiments [8]. A crucial step towards understanding the neuronal mechanisms underlying across-modality interactions in sensory perception is to record and characterize neuronal responses at the level of sensory-modality convergence. Using a previously established and reliable extracellular multiunit recording technique [9–14], we have access to that neuronal level in honeybees by recording mushroom body (MB) output neurons (MBONs). We use the term MBONs conditionally here as subpopulations of this class of neurons may also provide feedback (input) to the MB calyces or interconnect with other parts of the MB lobes in honeybees [15]. Similarly, in *Drosophila* neurons termed MBONs may not exclusively serve as output neurons as some MBON types interconnect within MB sub-regions [16].

However, MBONs, in the honeybee approximately 400 neurons [15], integrate activity from about 170 000 Kenyon cells (KCs), the intrinsic neurons of the MBs [17]. The MB calyx receives input from olfactory projection neurons (PNs) of the antennal lobe and visual PNs of the optic lobes and is organized in concentric layers, each of which is preferentially innervated by PNs of one modality. At this stage, sensory information converges with the reward pathway, which is facilitated by the ventral unpaired median neuron one of the maxillary neuromere (VUMmx1) [18]. The neurotransmitter octopamine mediates the reward [19,20]. Furthermore, a subpopulation of MBONs, the protocerebral calycal tract neurons (PCT), provide inhibitory feedback to the calyx [21]. The outermost layer (lip) receives olfactory input and visual PNs innervate the second concentric layer (collar) (e.g. [17,22–24]). A third layer (basal ring) receives layered input from both modalities. Thus, at the MB input level, both modalities are anatomically separated. The concentric organization of the MB calyx is maintained in distinct layers of the MB lobes [22]. Although it was reported that, for example, PCT neurons [25], or MB *β* neurons [26] (both can be seen as special clusters of MBONs), are sensitive to both odour and light stimuli, cross-modal integration in the honeybee has never been tested rigorously at the neurophysiological level.

We established an experimental design allowing characterization of activity in individual MBONs induced by olfactory, visual and olfactory–visual stimulation using two monochromatic light-emitting diode (LED) light sources (blue and green) and two odorants (farnesol and citronellol). The LEDs emitted wavelengths close to the absorbance maxima of the blue and green receptors [27] that were shown to be differentiated during a classical conditioning experiment [28]. The tested odorants were previously shown to evoke distinct antennal lobe activity in *Drosophila* [29] and in bumblebees [30]. Two olfactory–visual stimuli were presented as compounds—blue light + farnesol and green light + citronellol. Comparison of activity in MBONs evoked by unimodal and multimodal stimulation revealed, for the first time, evidence for cross-modal interaction in a group of multimodal MBONs showing an increased response rate when stimulated with the compound. Despite the substantial convergence from approximately 170 000 KCs to approximately 400 MBONs (425 : 1) [15,17], our results show that unimodal sensory input is still preserved by groups of unimodal MBONs responding to either light or odour stimulation.

## 2. Methods

### 2.1. Animals

Honeybee foragers (*Apis mellifera carnica*) from our departmental bee station at the University of Würzburg were captured and anaesthetized on ice before harnessing them in metal tubes such that only the mandibles, proboscis and antennae could freely move [31]. Heads were fixed with wax onto the metal tube, and the scapi of the antennae were fixed with low-melting-point wax on the head capsule. A small window was cut between the compound eyes. Head glands and trachea sacks were removed.

### 2.2. Odour stimulation

For olfactory stimulation, we used a custom-built olfactometer [11,30]. A constant air stream (1.5 m s^−1^) was provided via a Teflon tube (Ø 6 mm). Syringes (5 ml) were inserted into the tube to function as odour chambers. Farnesol and citronellol (Sigma-Aldrich Chemie GmbH) were diluted in paraffin oil (Sigma-Aldrich Chemie GmbH) to a 0.01 concentration (volume/volume). Filter papers (2 cm^2^) were soaked with 10 µl of odour solution and placed in the syringes. During the three seconds of stimulation, air volumes of only half of the syringes were injected into the air stream. An exhaust hood was placed behind the bee to quickly remove all odour molecules.

### 2.3. Light stimulation

**Figure 1.**
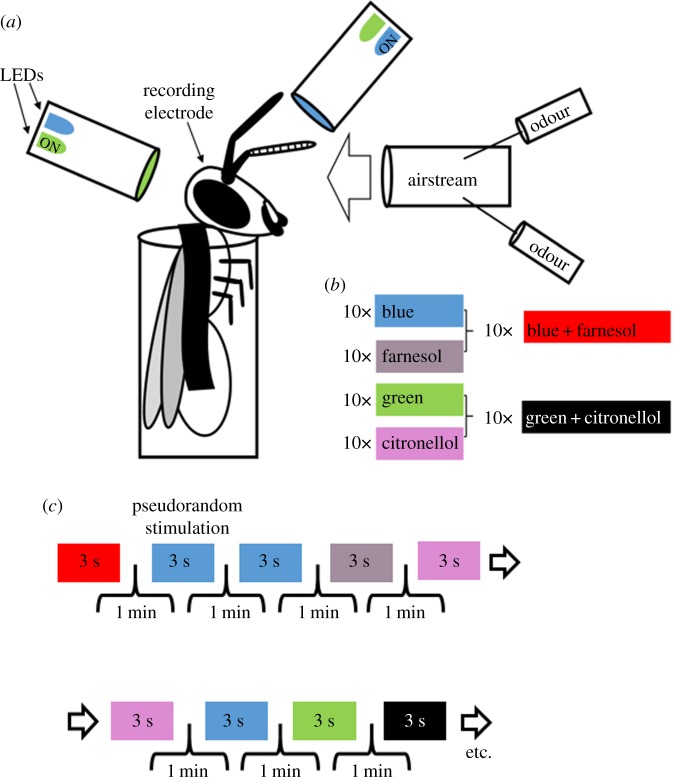
Odour, light and odour–light compound stimulation. (*a*) Honeybees were harnessed in metal tubes and stimulated with lights or odours while recording mushroom body output neurons (MBONs). (*b*) Two light stimuli (blue and green) and two odour stimuli (farnesol and citronellol), and two odour–light compound stimuli (blue + farnesol or green + citronellol) were repeatedly presented, 10 times each. (*c*) Stimuli were presented in a pseudorandomized sequence, meaning that a single stimulus was allowed to occur only two times in a row, but random. We used a one-minute inter-trial interval. Each stimulation lasted 3 s.

Two monochromatic LEDs (blue: 465 nm, intensity: 5.04 × 10^15^ photons cm^−2^ s^−1^; green: 525 nm, intensity: 3.93 × 10^15^ photons cm^−2^ s^−1^) were placed at the end of a transparent Plexiglas rod (diameter: 10 mm, length: 100 mm). Light transmission through the Plexiglas rod ensured diffusion before reaching the eye. Two Plexiglas rods were placed in a way to maximally illuminate each compound eye (figure 1*a*). The light intensity was measured by placing the light sensor of a radiometrically calibrated spectrophotometer (Jaz Spectrometer, Ocean Optics, Dunedin, FL, USA) at the position of the bee's compound eye.

### 2.4. Experiment

Blue light, green light, farnesol and citronellol were presented as single stimuli. Furthermore, we combined blue light with farnesol (blue + farnesol) and green light with citronellol (green+citronellol; figure 1*b*) as compound stimuli. Trial control software was used to synchronize data acquisition software and control stimulation via TTL-pulses (Cheetah 5, Neuralynx, Bozeman, MT, USA). All stimuli lasted for 3 s and were presented in pseudorandomized order, 10 times each using an inter-trial interval of 1 min (figure 1*c*).

### 2.5. Electrophysiology and data acquisition

We used a 16-channel digital data acquisition system from Neuralynx following previously established methods [10–13]. The extracellular recording electrode consisted of three micro wires (polyurethane-coated copper wire, 14 µm in diameter, Elektrisola, Escholzmatt, Switzerland). Neural activity was measured differentially from all pairwise combinations of the three wires using the Cheetah data acquisition software (Cheetah 5, Neuralynx) with a sampling rate of 30 kHz and high-pass filtered (greater than 600 Hz). A silver wire with a diameter of 25 µm (Nilaco, Tokyo, Japan) was used as reference electrode and inserted into the head capsule. All wires were connected to a head stage preamplifier (HS_16, Neuralynx).

The recording electrode was inserted at the ventral aspect of the vertical lobe at a depth between 100 and 200 µm. The depth control ensures that the recording tip was at least 100 µm away from other neuropils like the central complex or the lateral accessory lobe, which are located at a depth of at least 300 µm [32]. In that area, MBON neurites are up to approximately 10 µm [15] (see electronic supplementary material) thick and induce well-pronounced spike shapes in the differential recording channels which are distinct from other neurons in that area. In particular, KCs have much smaller neurite diameters (less than 0.5 µm) and therefore induce less pronounced action potential waveforms (for further details, see [10–12]). Following insertion, the hole in the head capsule was filled with two-component silicon (KWIK-SIL Sarasota, FL, USA) in order to prevent the brain from drying and to permanently anchor the electrode within the brain to avoid electrode drift.

### 2.6. Spike sorting

We applied a semi-automatic spike sorting technique (template-matching) provided by the Spike2 software (Cambridge Electronic Design, Cambridge, UK) on the differential recording channels as described in detail in [10,13]. In brief, we calculated the mean activity and standard deviation (s.d.) of the high-pass filtered channels and set the thresholds for detecting events at ±3 s.d. Threshold crossing events were used to compute templates of spike waveforms which were subsequently used to assign individual spikes. To control for single unit separation, we applied principal component analysis (PCA) of the detected waveforms. Single units had to show cluster separation after plotting their first three principal components. Furthermore, we plotted inter-spike interval (ISI) distributions of the units, which was allowed to be only above 1 ms. As the refractory period of neurons is larger than 1 ms, smaller ISIs would indicate double unit detection. All tools were provided in Spike2. In total, we could separate 92 single units recorded in 22 honeybees.

### 2.7. Data analysis

Data analyses were carried out with Matlab (MathWorks GmbH, Ismaning, Germany) and the FIND open source toolbox (http://find.bccn.uni-freiburg.de/) [33]. To obtain a time-resolved instantaneous mean firing rate for every unit, we used a convolution of the spike trains in each trial with a kernel to estimate firing rate [33,34]. As the neurons showed phasic tonic responses, we used an asymmetric kernel with the shape of the alpha function and applied an automatic method to determine the optimal kernel width (*τ*) as described by Nawrot and colleagues [34]*.* As the spontaneous baseline activity of the recorded units varied between 5 and 40 Hz which is consistent with intracellular recordings at this processing level [35], we applied a baseline correction for better visualization of the data. We averaged the baseline activity of 1000 ms before stimulus onset in every single unit and subtracted it from the unit's firing rate. A significant response in each unit was detected if the related stimulation led to a rate increase above 4 s.d. of the spontaneous activity. The baseline-corrected data were used to construct stimulus-dependent neuronal population vectors in the following way. For a given stimulus configuration *a* (odour, light or compound) and an ensemble of *n* neurons, we constructed the *n*-dimensional rate vector *v^a^* at each point in time during a 5000 ms time window (1000 ms before stimulus onset, 3000 ms during stimulation, 1000 ms following stimulus offset, compare heat maps in figure 3). The population vectors were further used in a PCA and to calculate the Euclidean distances (*L*^2^-Norm). For the latter, we performed a pairwise subtraction of the respective population vector couples (*v^a ^*− *v^b^*) as $\text{d}(t)={\left(\Sigma {\left(v_{i}^{a}(t)-v_{i}^{b}(t)\right)}^{2}\right)}^{1/2}$. Maximal firing rates were always extracted in the phasic response window (500 ms following stimulus onset).

### 2.8. Statistics

We used the Statistical Toolbox of Matlab. To test for statistical relevance of the stimulus-dependent maximal rate distributions and the absolute maximal rate difference distribution, we used a balanced one-way ANOVA followed by a two-sided Wilcoxon rank sum test.

## 3. Results

### 3.1. Olfactory, visual and olfactory–visual mushroom body output neurons

**Figure 2.**
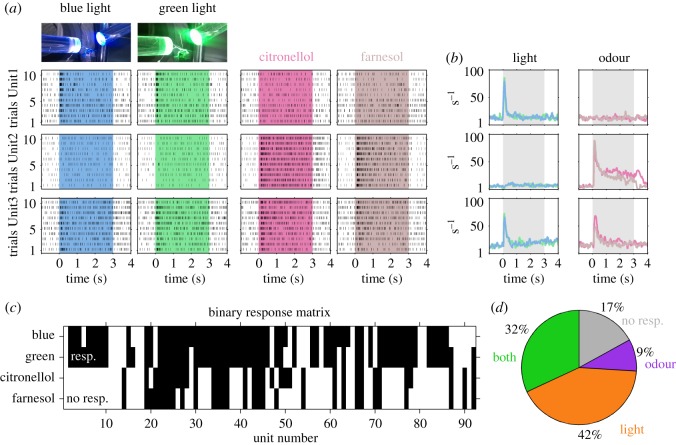
Single MBONs respond to either light, odour or both modalities. (*a*) The trial by trial resolved stimulus-dependent dot displays of three example units (rows) illustrate three different response behaviours. Unit1 (first row) responded reliably to blue light (first column) and green light (second column) in each single trial. Stimulation with citronellol (third column) and farnesol (fourth column) did not evoke responses in any trial. Unit2 (second row) behaved vice versa. Unit3 (last row) was reliably excited by both light and both odour stimuli. (*b*) The instantaneous firing rates of the 10 trials per stimulus were averaged for the three example units shown in (*a*) keeping the same odour and colour code. (*c*) Significant increase (black) in the averaged instantaneous firing rates (*b*) of each unit and type of stimulation is shown as a binary response matrix of the 92 recorded MBON-units. (*d*) The proportion of units responding to both modalities was 32%, to light stimulation only 42%, and 9% responded to odour stimulation only. A proportion of 17% was not sensitive to odour or light stimuli.

Although the MB calyx primarily receives multimodal input from olfactory and visual projection neurons, not all of the recorded MBONs were sensitive to both odour and light stimuli. We found three response categories as highlighted in the examples (figure 2). Unit1 responded to almost all presentations of green and blue light, but not to citronellol and farnesol. Unit2 showed the opposite and responded to both odour stimuli, but not light. Unit3 responded to both light and odour stimuli and was termed multimodal. These three types of MBON response categories were further analysed by calculating the averaged instantaneous firing rates across the 10 trials per stimulus in each unit (figure 2*b*). Interestingly, the response rate of individual units was very similar reaching comparable excitation levels and response dynamics, independent of stimulus identity.

We extracted the response properties of MBONs sensitive to both modalities by calculating a binary response matrix including all recorded units (figure 2*c*). This matrix comprises significant rate increases during stimulus presentations. From this matrix, we extracted the proportion of units sensitive to light or odour only, and units that were excited by both modalities (figure 2*d*). This revealed that 32% of the recorded units were multimodal MBONs sensitive to both visual and olfactory stimulation. The largest proportion (42%) of recorded MBONs were sensitive to light stimulation only, whereas 9% were sensitive to odour stimulation only. Interestingly 17% of the recorded MBONs were not responding to any of the stimuli tested.

### 3.2. Modality separation (categorization) rather than single stimulus separation

**Figure 3.**
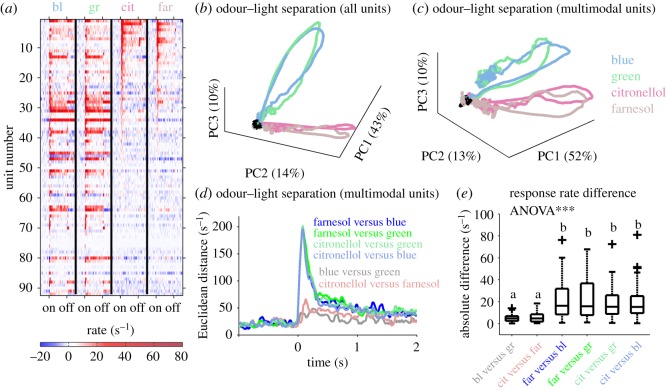
Odour–light separation by multimodal MBONs. (*a*) Averaged response rates for all 92 recorded units ordered by the strength of their citronellol induced responses. (*b*) The population response vector of all 92 units as shown in (*a*) was used in a PCA. The first three principal components (PC1–PC3) illustrate a separation between the two modalities, light and odour, whereas the two light and the two odour stimuli follow similar trajectories. Baseline activity is shown in black. (*c*) A similar picture emerges if only the multimodal units sensitive to both modalities (figure 2*d*) were used for PCA. (*d*) Euclidean distances between the different unimodal stimulus induced population activities of the 32% of multimodal units were calculated. The intramodal separation between blue and green light (grey), and citronellol and farnesol (pink), was equally distinct, whereas the intermodal separation between all odour–light combinations were drastically increased to a similar amount. (*e*) The absolute maximum response rate differences of the 32% multimodal units show a significantly different distribution (ANOVA; *p* < 0.001). A pairwise comparison of the absolute rate differences revealed a similar rate difference between the intramodal stimuli that was significantly different from the intermodal differences (Wilcoxon rank sum test; *p* < 0.001; different letters indicate significant differences).

A relatively large proportion of MBONs responded to combined odour and light stimuli. We therefore asked whether the two modalities might be separated at the population response level. As a first step, we focused on all recorded units (figure 3*a*) including the ones sensitive to one modality only and applied a PCA. The ensemble activity clearly separated the two modalities, but did not allow for separation of stimulus qualities within modalities (figure 3*b*). As modality separation could be mainly driven by unimodal units responding either to light or to odour, we excluded single modality-driven units from the analysis and applied the same procedure to multimodal units only. Interestingly, the result was very similar indicating that modality specificity was also encoded in the responses of multimodal MBONs (figure 3*c*). To further quantify modality separation by multimodal units, we calculated Euclidean distances between the responses to all stimulus pairs. The intramodal stimulus quality differentiation (blue versus green and citronellol versus farnesol) by the multimodal MBON population was comparable and rather low, whereas all intermodal stimulus pairs showed a much higher differentiation reaching comparably high amplitudes (figure 3*d*). The stimulus separation reflected in the absolute response rate difference distributions (figure 3*e*) was highly significant (ANOVA; *p* < 0.001). A pairwise comparison revealed a very similar picture—the intramodal differentiation was low and not distinct between the two wavelengths and the two odorant stimuli (Wilcoxon rank sum test, *p* > 0.05). However, they were significantly different from all intermodal differentiation pairs (Wilcoxon rank sum test, *p* < 0.001), which, compared to each other, reached the same level of differentiation (Wilcoxon rank sum test, *p* > 0.05).

### 3.3. Additive, but not linear computation of the multimodal compound

**Figure 4.**
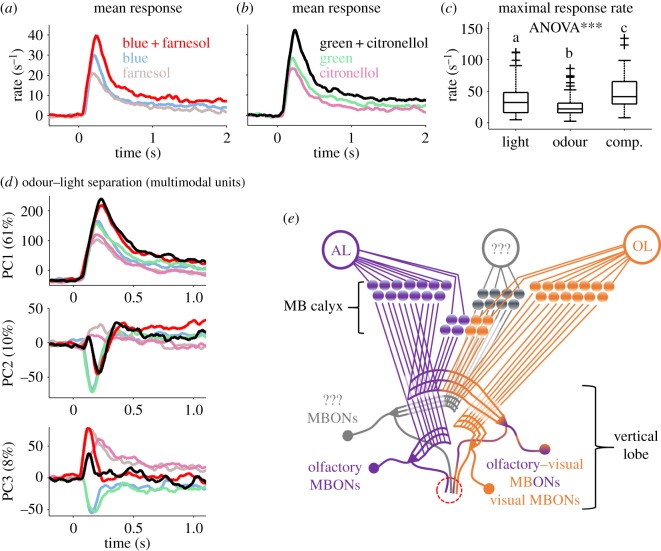
Additive odour–light integration separates the compound from the single modalities. (*a*) Averaged activity of multimodal units (32% of recorded units, figure 2*d*) in response to the single components light (blue) and odour (farnesol) and their olfactory–visual compound (blue + farnesol). The averaged response rate to compound stimulation is increased compared to the response to the single components. (*b*) Same as in (*a*) but for the single components light (green) and odour (citronellol) and their compound (green + citronellol). (*c*) Comparison between the odour, the light and the compound induced maximal response rate distribution. The different stimulus categories were significantly differentiated (ANOVA; *p* < 0.001, followed by a pairwise comparison using Wilcoxon rank sum test; *p* < 0.012; different letters indicate significant differences). (*d*) PCA on the multimodal MBON population. The first three principal components (PC1–PC3; *y*-axis) are shown over time (*x*-axis). PC1 explained 61% of variation in the data and separates three categories of stimuli: odour, light as well as the compound. Colour code is the same as in (*c*). In addition, PC2 (explaining 10% of variation) contrasts stimuli including a light component (negative values) from the two pure odours (positive values), whereas PC3 (explaining 8% of variation) contrasts pure light stimuli (negative values) from stimuli including odour-induced activity (positive values). (*e*) Schematic drawing of the hypothetical MB connectivity including purely olfactory (purple) as well as purely visually (orange) driven MBONs. In addition, a proportion of MBONs integrates olfactory and visual information across the MB input layers (purple–orange) separating the compound from its single components. A group of MBONs did not respond at all (grey). The red circle marked the recording position at the ventral aspect of the vertical lobe.

Next, we quantified how multimodal units compute simultaneous stimulation with both modalities by comparing the representation of both single modalities and both compounds (figure 4*a*,*b*). Interestingly, both combinations revealed similar results. The averaged activity was lowest for both odours, followed by the light-induced responses. The presentation of the olfactory–visual compound, in both cases, induced an increased response rate. As the maximal response rate distributions were similar for the two light stimuli, for the two odour stimuli and for the two odour–light compound stimuli (Wilcoxon rank sum test, *p* > 0.05), we merged the respective maximal response rate distributions for each stimulus class and tested odour versus light stimuli (Wilcoxon rank sum test, *p* = 0.012), odour-only versus compound stimuli (Wilcoxon rank sum test, *p* = 3.8 × 10^−7^), and light-only versus compound stimuli (Wilcoxon rank sum test, *p* = 0.009). All combinations were differentiated significantly (figure 4*c*; significance level after *post hoc* correction *p* < 0.016). As the increases in the response rates to stimulation with the olfactory–visual compounds do not represent a simple summation, they suggest a more complex type of nonlinear additive computation (figure 4*a–c*).

### 3.4. Multimodal compound stimuli form new categories at the ensemble level

The response rate increase we observed for the olfactory–visual compound stimulus (figure 4*a–c*) together with the observation that unimodal stimuli were separated with respect to their modality (figure 3*c–e*), but not their identity, led to the question if the olfactory–visual compound representation generates a new stimulus category at the MBON ensemble level. We therefore applied PCA including the single modality and the compound population activity vectors of the multimodal units. Visualizing the first three principal components (PC1–3) separately over time (figure 4*d*) illustrates that at the MBON level both olfactory–visual compound stimuli open a new category by being clearly separated from the single modalities. However, they were not separated from each other, as shown most distinctly by PC1 (figure 4*d*). In addition, PC2 and PC3 extracted different modality (‘category’) features. PC2 contrasted stimuli including light information from those containing pure odours, whereas PC3 contrasted activity induced by pure light from stimuli including an odour component. Altogether, this suggests that the MBON level represents distinct categorizations between modalities (olfaction and vision) and their compound stimuli.

## 4. Discussion

### 4.1. Neuronal amplification of the compound by olfactory–visual interactions

In a noisy room, it is advantageous to have visual contact with someone whose words we try to understand. This so-called ‘cocktail party effect’ highlights an important feature of multisensory integration—the ability of a given sensory modality to interact with another by enhancing or suppressing its sensation [36]. In humans, cross-modal interactions can also be highlighted by the phenomenon that the nose smells what the eye sees [37]. Our analyses of honeybee MBONs revealed a neuronal correlate of such interactions. Similar to the olfactory tubercle of rats, where a proportion of multi-sensory neurons (sensitive to sound and odour) showed additive responses to the compound [38], we observed an additive representation of the olfactory–visual compound compared to its single components. The response rates of the compound were always smaller than the sum of the response rates with modalities presented alone. We therefore suggest an additive, but not linear neuronal computation, which we interpret as a neuronal amplification (enhancement) of olfactory and visual input when occurring as a compound. This could be interpreted as the neuronal substrate of a ‘flower party effect’ in honeybees.

### 4.2. Rapid categorization of sensory information

In vertebrates and invertebrates, receptor neuron activity needs to be transformed at different processing levels to encode relevant environmental features. This is true for both the olfactory [39–42] and the visual [43–45] neuronal pathways. After unimodal processing, the different modalities need to interact to form a common percept of the environment at high-order multimodal integration centres like the insect MBs (e.g. [15,23,46,47]). To evoke a fast and adequate behavioural response, information needs to be categorized rapidly (good, bad, animal, plant, flower, tree etc.). Humans, for example, need less than 30 ms exposure time for categorization of an animal from a series of random pictures [48]. An adequate response including all the necessary motor activity was established approximately 400 ms later. In honeybees, an odour stimulus with only 200 ms duration can be associated with a reward [49]. Moreover, differences in the onset of odorant stimuli of only 6 ms were shown to be sufficient for odour–object categorization [50]. Although these examples deal only with one modality, they underline the high speed of stimulus categorization. Therefore, neuronal representations of sensory input at high-order integration centres need to be established rapidly after stimulus onset. Simultaneous recordings from MBONs and PNs of the antennal lobe recently showed that MBONs reached maximal odour induced activity only approximately 70 ms after stimulus onset, at a time when encoding at the level of the antennal lobe has not reached maximal stimulus separation [11]. This rapid activation of MBONs before completing antennal lobe computation is probably the reason why MBONs in unconditioned animals did not show significant odour tuning as was revealed when testing ten different odorants [10]. However, this fast representation of the category ‘odour’ allows the higher order brain centre to trigger appropriate neuronal processes including identification of the exact odour identity at the AL level. Another category of stimuli might be present after classical conditioning, when single MBONs become recruited to respond to the reward-associated stimulus encoding the valence [10,12], which is in line with the general concept of valence encoding in MBONs [51] which can also be found in *Drosophila* [52]. As MBONs in unconditioned honeybees show a broad odour tuning [10], we expect the same unspecialized tuning in response to light stimuli which is supported by our results. Taken together, the MB has the capacity to integrate across modalities to encode the valence to provide rapid environmental stimuli representations allowing fast downstream decision processes. We found a separation in three broad categories of stimuli (odour, light and odour + light) at the level of MBONs, which were not separated regarding stimulus quality within each category. We therefore propose that the MB rapidly integrates modalities to form stimulus categories that allow fast activation of memory or other neural processes necessary to evaluate the appropriate behaviour.

### 4.3. Different MBON response categories may correspond to morphologically distinct MBON clusters

By stimulating with light of different wavelengths, two odorant stimuli, and two olfactory–visual compound stimuli, our recordings and analyses revealed four distinct MBON categories (figures 2*d* and 4*e*): MBONs sensitive to light only (c1), MBONs sensitive to odour only (c2), multimodal MBONs sensitive to both modalities (c3), and MBONs that did not respond at all (c4). We can exclude that c3 might be caused by sorting and extracting spikes that might derive from two neurons. First, during compound stimulation, the two neurons' bursts would occur simultaneously. Consequently, ISIs < 1 ms would show up, which was not allowed to occur in a single unit following our sorting criteria. Second, simultaneous bursting of two neurons would most likely result in coincident action potentials. With extracellular recordings, in that case, the voltages of two action potentials would sum up resulting in an additional waveform which would be detected as an additional unit of spikes. This additional unit would be sensitive to the olfactory–visual compound only. As such a response behaviour never occurred, we are very confident that all separated units can be truly assigned to the activity of single neurons.

Given the electrode insertion position at the ventral vertical lobe at a depth between 100 and 200 µm, we potentially recorded from MBONs associated with the A1, A2, A4, A5 and A7 extrinsic neuron clusters (nomenclature by [15]; for details see electronic supplementary material). Therefore, each of the four response categories might be related to one of the five cell clusters. The non-responding MBONs (c4) might include two clusters, for example MBONs responsive to additional modalities relayed to the MB input (e.g. tactile and gustatory [53]) and MBONs that are initially silent and eventually become recruited to encode an odour reward association after memory formation [10,12]. Although this match between MBON responses and anatomical MBON clusters is speculative, it receives support by the internal structure of the MB calyx with distinct layers of KC output in the MB vertical lobes [22] and MBON synaptic input. Mobbs suggested a three-layered organization of the vertical lobe [17], each of which matched to one of the MB calyx input layers (lip, collar, basal ring). This was later extended to a fourth layer in the honeybee MB vertical lobe, the gamma-lobe [23]. Besides the mainly unimodal lip (olfaction) and collar (vision) (e.g. [17,22–24]), the basal ring receives olfactory, visual and potentially projections from other modalities segregated into different concentric layers [47], and KC dendrites may span across several of these layers [22]. Although physiological proof is still lacking, basal-ring KCs (inner compact, class I KCs), therefore, might potentially provide multimodal input to MBONs responding to both modalities, whereas MBONs receiving input from KCs with dendrites limited to the lip and collar (class I non-compact KCs) might be restricted to only one modality.

Anatomically, the MB calyx lip and collar are equally prominent MB input compartments in the honeybee [17]. However, we found that only 9% of the recorded MBONs respond to odour only, whereas 42% responded to light only, and 32% to the compound stimulus. While a total of 92 MBON units sampled from a heterogeneous group of foragers with unknown previous experience are still low, this might indicate that especially olfaction, to a large extent, may already be combined with visual information at this still early processing level providing an additional and highly important stimulus category. Furthermore, the MBON categories are likely to be affected by plasticity, in particular related to learning and memory, as KCs exhibit non-associative and associative plasticity [54] and potentially have the capacity to modulate [55] or even recruit initially silent MBONs during memory retention at their output synapses [10,12].

## Supplementary Material

Recording mushroom body output neurons at the ventral vertical lobe of the honeybee

## Data accessibility

The dataset with all recorded single units is made publicly available on the database of the German Neuroinformatics Node (www.g-node.org; doi:10.12751/g-node.d919d4).
