# Supplementary material for: Multimodal integration and stimulus categorization in putative mushroom body output neurons of the honeybee
Source: R Soc Open Sci. 2018 Feb 21;5(2):171785. doi: 10.1098/rsos.171785 (PMC5830775; doi:10.1098/rsos.171785)
Supplement: Recording mushroom body output neurons at the ventral vertical lobe of the honeybee [file rsos171785supp1.pdf]

Multimodal Integration and Stimulus Categorization in Putative Mushroom Body Output Neurons of the Honeybee

Martin F. Strube-Bloss and Wolfgang Rössler*

Behavioral Physiology and Sociobiology, Biocenter University of Würzburg, Am Hubland, 97074 Würzburg, Germany

***Corresponding author:** e-mail: martin.strube-bloss@uni-wuerzburg.de

Supplementary Material

S1: recording mushroom body output neurons of the ventral vertical lobe

The applied stimulation protocol includes 10 repetitions of two different light, two different odour and two different odour-light compound stimuli. To avoid adaptation effects we presented the stimuli with a one minute inter trial interval pseudorandomized. This means we needed at least 60 minutes of stable recordings. Intracellular recordings, because of recording time limitation, were no option. However, to record from mushroom body output neurons (MBON) in honeybees over such a long time period, we used a well-established extracellular long term recording technique. Using this approach stable measurement of neuronal spiking activity for many hours has been documented for antennal lobe projection neurons [1-3] as well as MBONs [3-7] in the honeybee – even in simultaneous recordings at two locations. After opening head capsule and removing the glands on top of the brain the different neuropils can be seen. The vertical lobes can be identified (even at low magnification) as dark shaded circular areas, and, therefore, are easily accessible (supplementary figure S1).

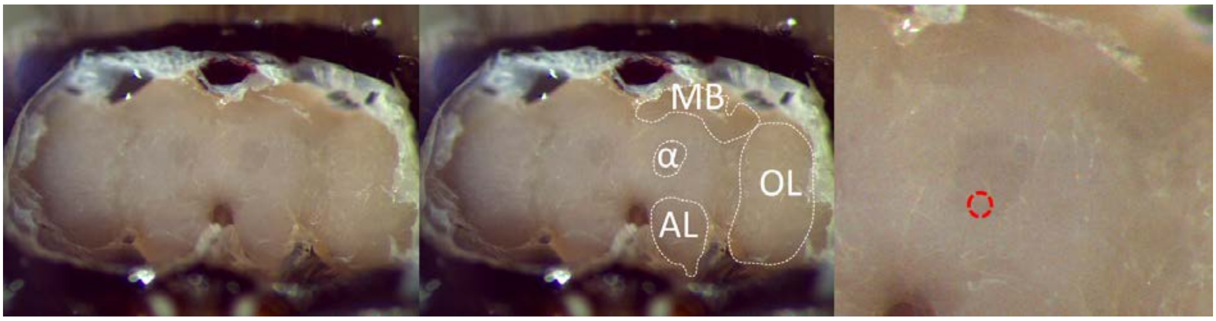

Supplementary figure S1: identifying the vertical (α) lobe after opening the head capsule. *Other neuropils like the mushroom body calyx (MB), the optic lobes (OL) and the antennal lobe (AL) can be easily seen under a conventional light microscope. The vertical lobe has a diameter of about 200 µm. We positioned the three channel differential recording electrode which has a total diameter of about 30 µm at the ventral aspect (6-7 o'clock position) of the vertical (α) lobe (red circle).*

We positioned the electrode at the surface of the ventral aspect of the vertical (α) lobe (6-7 o'clock position) and vertically penetrated the lobe until the electrode picked up well pronounced action potentials, usually at a depth between 100-200 µm, which is the depth were most of the relatively thick MBON-neurites overlap with Keyon cell axons [8]. At this depth the recording tip was at least 100 µm away from other neuropils like the central complex or the lateral accessory lobe which border at a depth of 300 µm (see standard brain atlas of the honeybee: http://www.neurobiologie.fu-

berlin.de/beebrain). Neurites of MBONs in this region have diameters of up to 10 µm, as illustrated by the example we adopted from Brandt and colleagues (2005) [9]. Following the nomenclature introduced by Rybak and Menzel [8] the recorded neurons can be related to the A1, A2, A4, A5 and A7 cluster of the mushroom body output neurons (supplementary figure S2). Because of their big axo-dendrites MBONs in that area induce well pronounced spike shapes in our differential recording channels (supplementary figure S3), which can be reliably sorted to obtain single unit activity (cp. supplements in [1, 7]). In contrast, other neurons in that area, in particular Kenyon cells, have neurite diameters of <0.5 µm and, therefore, induce much smaller action potential waveforms as shown in the supplements of an earlier publication [7]).

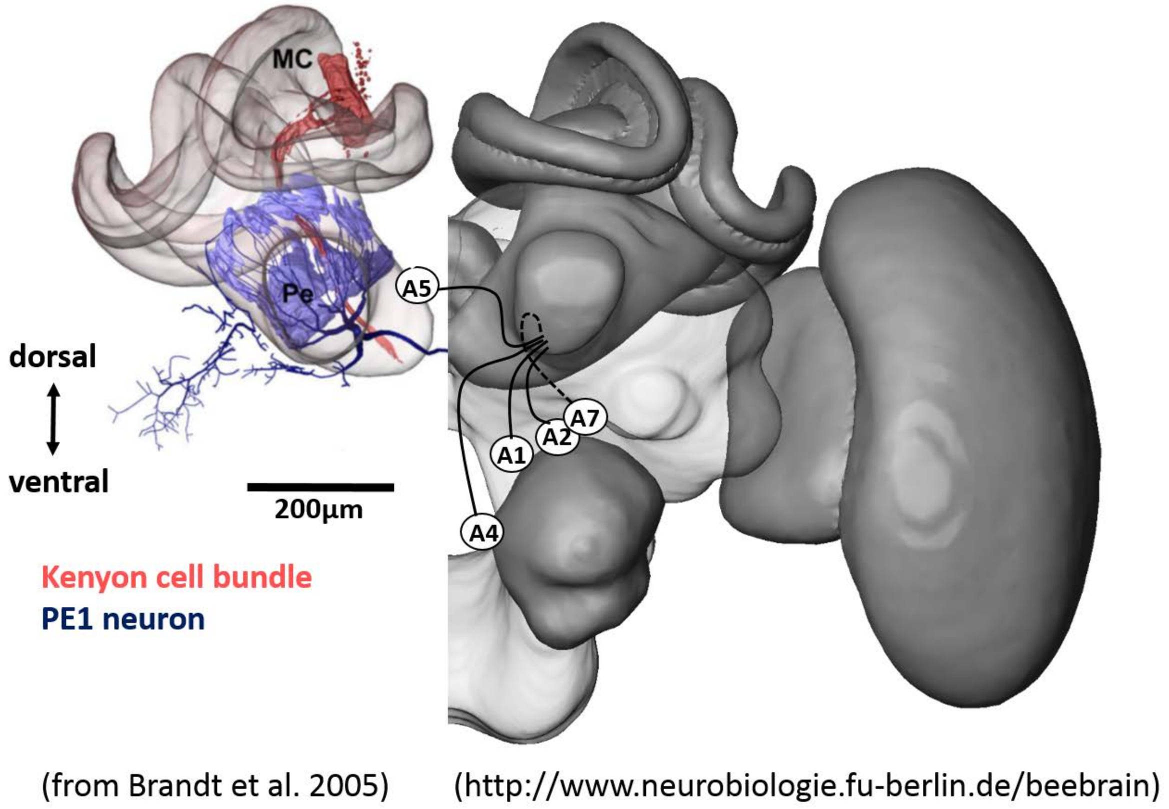

Supplementary figure S2: recording extracellularly from mushroom body output neurons*. left: The pedunculus output neuron #1 (PE1, blue) is shown as a representative of target neurons of the present study [9]. Note, the neurite at the ventral vertical lobe is bigger than the whole Kenyon cell bundle (red), which is the reason why extracellular recorded actionpotentials are rather destinctive. right: 3D image of the honeybee brain (http://www.neurobiologie.fu-berlin.de/beebrain). Neurons at the recording site can be related to the A1, A2, A4, A5 and A7 cluster of mushroom body output neurons (after Ryback and Menzel 1993).*

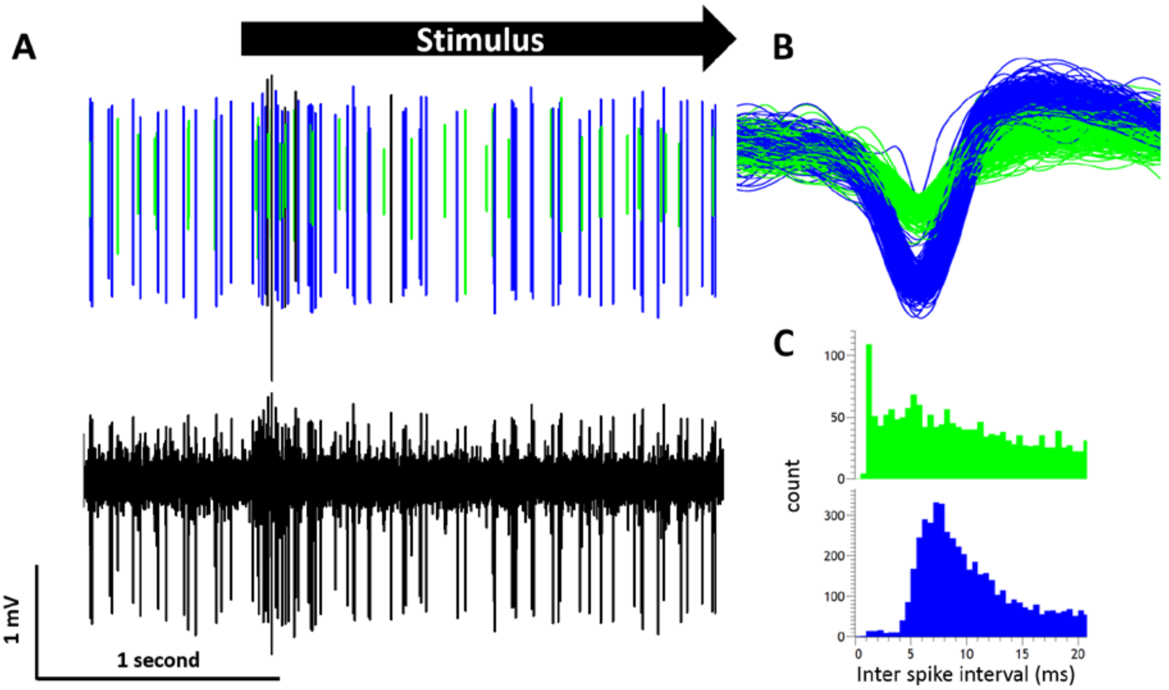

Supplementary figure S3: signal to noise ratio allows single unit extraction. A| *Raw data (bottom) and extracted single spike events (top) of two different units (blue and green).* ***B|*** *Waveform overdraw of the two single units (same color code as in A).* ***C|*** *interspike intervall (ISI) distribution of the two extracted units. Note, both units show ISIs biger than 1ms.*

Supplementary References

1. Brill MF, Reuter M, Rössler W, Strube-Bloss MF. 2014. Simultaneous long-term recordings at two neuronal processing stages in behaving honeybees. *J Vis Exp* **89**: 1-13. (doi:10.3791/51750)
2. Strube-Bloss MF, Brown A, Spaethe J, Schmitt T, Rössler W. 2015. Extracting the Behaviorally Relevant Stimulus: Unique Neural Representation of Farnesol, a Component of the Recruitment Pheromone of Bombus terrestris. *PLoS One* **10**: 1–16. (doi:10.1371/journal.pone.0139296)
3. Strube-Bloss MF, Herrera-Valdez MA, Smith BH. 2012. Ensemble response in mushroom body output neurons of the honey bee outpaces spatiotemporal odor processing two synapses earlier in the antennal lobe. *PLoS One* **7:** 1–16. (doi:10.1371/journal.pone.0050322)
4. Strube-Bloss MF, Nawrot MP, Menzel R (2016) Neural correlates of side-specific odour memory in mushroom body output neurons. *Proc. R. Soc. B* 283: 20161270. http://dx.doi.org/10.1098/rspb.2016.1270
5. Okada R, Rybak J, Manz G, Menzel R. 2007. Learning-related plasticity in PE1 and other mushroom body-extrinsic neurons in the honeybee brain. *J Neurosci* **27:** 11736–11747. (doi:10.1523/jneurosci.2216-07.2007)
6. Farkhooi F, Strube-Bloss M, Nawrot M. 2009. Serial correlation in neural spike trains: Experimental evidence, stochastic modeling, and single neuron variability. *Phys Rev E* **79**: 1-10. (doi:10.1103/physreve.83.050905)
7. Strube-Bloss MF, Nawrot MP, Menzel R. 2011. Mushroom body output neurons encode odor-reward associations. *J Neurosci* **31**: 3129–3140. (doi:10.1523/jneurosci.2583-10.2011)
8. Rybak J, Menzel R. 1993. Anatomy of the Mushroom Bodies in the Honey Bee Brain: The Neuronal Connections of the Alpha-lobe. *J Comp Neurol* **334**: 444–465. (doi:10.1002/cne.903340309)

9. Brandt R, Rohlfing T, Rybak J, Krofczik S, Maye A, Westerhoff M, Hege H-C, Menzel R. 2005 Three-dimensional average-shape atlas of the honeybee brain and its applications. *The Journal of comparative neurology* **492**, 1–19. (doi:10.1002/cne.20644)
